# Cardiorespiratory fitness and lifestyle on severe COVID-19 risk in 279,455 adults: a case control study

**DOI:** 10.1186/s12966-021-01198-5

**Published:** 2021-10-19

**Authors:** Elin Ekblom-Bak, Daniel Väisänen, Björn Ekblom, Victoria Blom, Lena V. Kallings, Erik Hemmingsson, Gunnar Andersson, Peter Wallin, Jane Salier Eriksson, Tobias Holmlund, Magnus Lindwall, Andreas Stenling, Amanda Lönn

**Affiliations:** 1 Department of Physical Activity and Health, The Swedish School of Sport and Health Sciences, PO Box 5626, 114 86 Stockholm, Sweden; 2 Research Department, HPI Health Profile Institute, PO Box 35, 182 11, Danderyd, Sweden; 3 Department of Neurobiology, Care Sciences and Society, Division of Physiotherapy, Karolinska Institute, 141 83 Stockholm, Sweden; 4 Department of Psychology, University of Gothenburg, 405 30 Gothenburg, Sweden; 5 Department of Psychology, Umeå University, 901 87 Umeå, Sweden; 6 Department of Sport Science and Physical Education, University of Agder, 4630 Kristiansand, Norway; 7 Women’s Health and Allied Health Professionals Theme Medical Unit Occupational Therapy and Physiotherapy, Stockholm, Sweden

**Keywords:** Cardiorespiratory fitness, Lifestyle, Obesity, Socioeconomics, Severe acute respiratory syndrome coronavirus 2

## Abstract

**Background:**

The impact of cardiorespiratory fitness (CRF) and other lifestyle-related factors on severe COVID-19 risk is understudied. The present study aims to investigate lifestyle-related and socioeconomic factors as possible predictors of COVID-19, with special focus on CRF, and to further study whether these factors may attenuate obesity- and hypertension-related risks, as well as mediate associations between socioeconomic factors and severe COVID-19 risk.

**Methods:**

Out of initially 407,131 participants who participated in nationwide occupational health service screening between 1992 and 2020, *n* = 857 cases (70% men, mean age 49.9 years) of severe COVID-19 were identified. CRF was estimated using a sub-maximum cycle test, and other lifestyle variables were self-reported. Analyses were performed including both unmatched, *n* = 278,598, and sex-and age-matched, *n* = 3426, controls. Severe COVID-19 included hospitalization, intensive care or death due to COVID-19.

**Results:**

Patients with more severe COVID-19 had significantly lower CRF, higher BMI, a greater presence of comorbidities and were more often daily smokers. In matched analyses, there was a graded decrease in odds for severe COVID-19 with each ml in CRF (OR = 0.98, 95% CI 0.970 to 0.998), and a two-fold increase in odds between the lowest and highest (< 32 vs. ≥ 46 ml·min^−1^·kg^−1^) CRF group. Higher BMI (per unit increase, OR = 1.09, 1.06 to 1.12), larger waist circumference (per cm, OR = 1.04, 1.02 to 1.06), daily smoking (OR = 0.60, 0.41 to 0.89) and high overall stress (OR = 1.36, 1.001 to 1.84) also remained significantly associated with severe COVID-19 risk. Obesity- and blood pressure-related risks were attenuated by adjustment for CRF and lifestyle variables. Mediation through CRF, BMI and smoking accounted for 9% to 54% of the associations between low education, low income and blue collar/low skilled occupations and severe COVID-19 risk. The results were consistent using either matched or unmatched controls.

**Conclusions:**

Both lifestyle-related and socioeconomic factors were associated with risk of severe COVID-19. However, higher CRF attenuated the risk associated with obesity and high blood pressure, and mediated the risk associated with various socioeconomic factors. This emphasises the importance of interventions to maintain or increase CRF in the general population to strengthen the resilience to severe COVID-19, especially in high-risk individuals.

**Supplementary Information:**

The online version contains supplementary material available at 10.1186/s12966-021-01198-5.

## Background

The coronavirus disease 2019 (COVID-19) has become a public health emergency worldwide. Among approximately one million laboratory confirmed COVID-19 cases in Sweden, over 57,000 have been hospitalised and more than 14,000 COVID-19 related deaths have been confirmed (up until May 15^th^, 2021). Mechanisms explaining a higher vulnerability to severe COVID-19 have been linked to inflammation characterized by increased levels of several pro-inflammatory cytokines and the inflammasome [1]. In turn, this has resulted in an inter-individual variation in severity of COVID-19 infection, so that, for example, older age, male gender and one or more comorbidities have been associated with increased risk for hospitalization and mortality due to COVID-19 [2–4]. Also, lifestyle-related factors have been linked to COVID-19 severity. Overall/central obesity and hypertension were the first and most frequently reported factors found to be more prevalent in individuals who were hospitalized or died due to COVID-19 [5–8]. In later papers, physical inactivity has been linked to severe COVID-19 risk [9, 10] and in a small sample of men and women, lower cardiorespiratory fitness (CRF) has also been associated with a higher risk of hospitalisation for COVID-19 [11].

Only a few studies have investigated the importance of lifestyle factors on severe COVID-19 risk, and it is plausible that, based on previous knowledge, a healthy lifestyle before infection may reduce the risk of severe COVID-19. A positive impact on inflammation and the immune system is one possible mechanistic pathway [12, 13], as low-grade inflammation is considered to be a strong causal factor for chronic diseases such as cardiovascular disease and cancer [14]. Also, the possible impact of a healthy lifestyle on other risk factors, such as overweight/obesity and hypertension [6, 15], may induce protection against severe COVID-19, and regular physical activity (PA) has been suggested as a protective non-pharmacological tool against COVID-19 [12, 16]. However, the research underpinning these assumptions is limited, as are previous studies looking at the importance of and the interaction between different lifestyle-related factors for COVID-19 severity.

Apart from the above predictors, lower socioeconomic status (assessed as, for example, educational level, income or area of residence) has been related to more severe COVID-19 [2, 17, 18]. The subsequent severity of the COVID-19 infection may, however, not only be explained by structural socioeconomic factors, but also by more unfavourable lifestyle habits and poorer health status before infection in individuals with lower socioeconomic status [19, 20]. If and how lifestyle mediates some of the associations seen between socioeconomic factors and severe COVID-19 has not yet been investigated.

The identified knowledge gaps above are addressed in the present study, with the main aim being to study a wide span of lifestyle-related and socioeconomic factors as potential predictors of severe COVID-19, and with special focus on CRF. Secondary aims are to study whether CRF may attenuate obesity- and hypertension-related risk of severe COVID-19, and whether lifestyle-related factors mediate the associations between socio-economic factors and severe COVID-19 risk. The hypotheses are that lifestyle-related (in particular CRF) and socioeconomic factors can predict severe COVID-19, and that variations in lifestyle-related factors mediate a large proportion of the risk of severe COVID-19 associated with socioeconomic factors.

## Methods

The study is a nested case–control study based on data from the Health Profile Assessment (HPA) database (www.hpi.se). HPAs have been carried out in health services all around Sweden since the middle of the 1970s and is offered to all employees working for a company or an organization connected to occupational or health-related services. An HPA includes a questionnaire about lifestyle and health experiences, measurements of anthropometrics and blood pressure, estimations of maximal oxygen consumption (VO_2_max) from a submaximal cycle ergometer test, and a person-centred dialogue with a HPA coach.

In February 2021, a total of 407,131 HPAs between 1992 and 2020 were available in the database, and the database was linked to national registries with data on severe COVID-19 (defined as hospitalization, intensive care or death due to COVID-19) using the unique Swedish personal identity number. A total of 857 (0.2%) confirmed cases with severe COVID-19 were identified, including COVID-19 hospitalization (*n* = 547, 0.1%), intensive care (*n* = 172, 0.04%) and death (*n* = 138, 0.03%). Controls were recruited from the same HPA database. All deceased controls before 2019–12-31 according to the national cause of death registry were excluded. To minimize internal drop-out, only participants without severe COVID-19 and with valid data on sex, age, educational level, CRF, body mass index (BMI), exercise and smoking were eligible as controls (a total of *n* = 278,598). Eligible controls were in general more often women, older, and had higher CRF and lower BMI (see overview of included and excluded participants in Additional file 1). The study was approved by the ethics board at the Stockholm Ethics Review Board (Dnr 2020–02,727). Informed consent was obtained from the participants prior to participation in the HPA. It was not possible to involve participants or the public in the design, conduct, reporting or dissemination plans of our research, due to its retrospective design.

### Estimation of VO_2_max

Measurement of CRF as actual maximal oxygen uptake (VO_2_max), using a graded test to exhaustion, is limited in the general population for numerous reasons including health risks in non-athletic populations and dependence on laboratory equipment and expertise. Therefore, CRF was assessed as estimated VO_2_max (estVO_2_max) from the standardized submaximal Åstrand cycle ergometer test in L·min^−1^ and also expressed in relative values (ml·min^−1^·kg^−1^) [21]. Previous validation studies on adult population samples show small and non-significant mean differences on a group level (− 0.07 L·min^−1^ 95% CI − 0.21 to 0.06) between estVO_2_max from the Åstrand protocol and direct measured VO_2_max during treadmill running with an absolute error and coefficient of variance similar to other submaximal tests (SEE = 0.48 L min^−1^, CV = 18.1%) [22]. To minimize well-known errors with submaximal testing, participants were requested to refrain from vigorous activity the day before the test, consuming a heavy meal and smoking/using snuff three hours and one hour respectively before the test, as well as avoiding physiologic and emotional stress prior to the test. The participant cycled on a calibrated ergometer at an individually adapted submaximal work rate (aiming at a rate of perceived exertion of “Somewhat hard”, 13–14, on the Borg RPE scale) for 6 min to achieve a steady-state pulse assessed during the last minute of cycling. Using the steady- state pulse and the work rate, VO_2_max was estimated from a sex-specific nomogram, with corresponding age-correction factors [21].

### Other measurements

Body mass was assessed in light-weight clothing using a calibrated scale and to the nearest 0.5 kg. Body height was assessed to the nearest 0.5 cm using a wall-mounted stadiometer. Body mass index (BMI) (kg·m^−2^) was subsequently calculated. Central obesity was assessed as waist circumference and measured to the nearest 0.5 cm with a tape measure at the midpoint between the top of the iliac crest and the lower margin of the last palpable rib in the mid axillary line after normal exhalation. Systolic and diastolic blood pressure (BP) were measured manually by the standard auscultation method in the seated position after 20 min of resting.

### Self-reported and register data

Exercise, commute type, physical work situation, diet habits, alcohol habits, smoking, overall stress, and perceived health were self-reported (see Additional file 2). Highest educational attainment, occupation, income, civil status, and data on country of birth at the time for the HPA were obtained from Statistics Sweden by linking of the participants’ personal identity numbers. Educational attainment was collected from the Swedish education nomenclature 2000 and was categorised into three categories: Elementary school, High school/Vocational education, and University. Each occupation is labelled and defined by a four-digit code according to the Swedish Standard Classification of Occupation [23]. In the present study, occupations were further aggregated according to the first digit into white-collar high-skilled (Major group 1–3), white-collar low-skilled (Major group 4–5), blue-collar high skilled (Major group 6–7) and blue-collar low-skilled (Major group 8–9). For a more detailed description see publication [24] by Väisänen et al. Total income from employment for the specific year was derived from the Income and Taxation Register. Cases of chronic disease before 2020 were ascertained through the Swedish national patient registry using ICD-coding; C00-D48 to define tumour disease, E10 to E14 for diabetes, I10 to I15 for hypertension, I26 to I28 + J44 to J45 for lung disease and I20 to I25 + I30 to I52 + I60 to I69 for cardiovascular disease.

### Severe COVID-19 surveillance

The main outcome in the present study is severe COVID-19, which is defined as either hospital admission, admission to ICU and/or death due to COVID-19. Participants’ personal identity numbers were linked to national registers. Data on hospitalization was obtained from the Swedish National Patient Register, data on intensive care was obtained from the Swedish Intensive Care Registry, and data on death was obtained from the National Cause of Death Register.

### Statistics

Main analyses include cases with sex- and age-matched controls, as the risk of severe COVID-19 is strongly associated with male gender and higher age. Sensitivity analyses using unmatched controls are included in supplementary material (see Additional file 3). For matched analyses, each case was matched to four eligible controls out of the 278,598 eligible controls in the HPA database, with no tolerance in variation between sex or age (per year) between the case and the controls and without replacement of controls in the sampling. For unmatched analyses all eligible controls were used. To compare descriptive characteristics of cases and controls, paired t-test (continuous data), Cochran's Q test (nominal data) and Related-Samples Friedman's Two-Way Analysis of Variance by Ranks (categorical data) were used, and effect sizes as Cohen´s d is presented for continuous data. To compare descriptive characteristics between cases of severe COVID-19, chi-square test of independence with false discovery rate correction for multiple testing (categorical data) and ANCOVA (continuous data) were used. Logistic regression modelling was used to calculate odds ratio (OR) with 95% confidence intervals (95% CI) for different predictors of serve COVID-19. Three models were used and adjusted for an increasing number of variables (see under each table/figure). Model 1 included sex, age and performed year of HPA. Model 2 additionally adjusted for educational level, civil status and country of birth, and Model 3 also adjusted for CRF, BMI, number of chronic diseases, exercise habits, smoking and overall stress. Due to internal drop-out for variables included in Model 2 and 3, two Model 1’s are presented; one Model 1 with OR and 95% CI including all available individuals (labelled *Non-complete data* in the tables, referred to as *Model 1-nc*), and a second Model 1 including only individuals with complete data for all adjusting variables (labelled *Complete data for all adjusting variables* in the tables, referred to as *Model 1-c*). Further, BMI, waist circumference, blood pressure, estVO_2_max and income from employment were analyzed as continuous variables as well as after aggregation; BMI was aggregated into normal weight < 25, overweight 25–29.9, obesity 30–34.9 and severe obesity ≥ 35 kg·m^−2^; large waist circumference was defined as ≥ 88 cm for women and ≥ 102 cm for men, (both BMI and waist circumference were categorized according to recommendations by the world health organization [25]); high systolic and diastolic BP were defined as ≥ 140 mmHg and ≥ 90 mmHg, respectively; estVO_2_max was arbitrarily categorised based on multiples of one METs (3.5 ml·min^−1^·kg^−1^) into < 25 (very low), 25- < 32 (low), 32- < 46 (moderate) and ≥ 46 (high) ml·min^−1^·kg^−1^; and total income from employment into quartiles, percentile 25 = 281,143, percentile 50 = 362,718, percentile 75 = 479,764 Swedish crowns. All above analyses were performed using IBM SPSS (V.26.0.0.1) and Jamovi (The jamovi project (2021) Version 1.6. retrieved from https://www.jamovi.org). Marginal effects plots for severe COVID were calculated by setting the covariates at a mean (for continuous variables) or average (for factor variables) level while varying the focal variables, using R (R Core Team, 2021) and the packages Tidyverse [26] and ggeffects [27]. Mplus version 8.6 [28] was used to estimate Bayesian parallel mediation models linking socioeconomic indicators to severe COVID-19 via multiple mediators (Fig. 2). Separate models were estimated for each socioeconomic indicator. The highest socioeconomic category was used as the reference category in each model. CRF, BMI, exercise, and stress were treated as continuous variables whereas smoking was dichotomized into daily smoker or never/seldom smoking. We used the proportion of the total effect that is mediated as an effect size measure [29]. The proportion mediated by each mediator was calculated by dividing the specific indirect effect by the total effect (*a*_*1*_*b*_*1*_) / (*a*_*1*_*b*_*1*_ + *a*_*2*_*b*_*2*_ + *a*_*3*_*b*_*3*_+ *a*_*4*_*b*_*4*_+ *a*_*5*_*b*_*5*_+ *c’*). The total proportion mediated was calculated by dividing the sum of the indirect effects by the total effect (*a*_*1*_*b*_*1*_ + *a*_*2*_*b*_*2*_ + *a*_*3*_*b*_*3*_+ *a*_*4*_*b*_*4*_+ *a*_*5*_*b*_*5*_) / (*a*_*1*_*b*_*1*_ + *a*_*2*_*b*_*2*_ + *a*_*3*_*b*_*3*_+ *a*_*4*_*b*_*4*_+ *a*_*5*_*b*_*5*_+ *c’*). Models were estimated using four Markov chain Monte Carlo chains and a minimum of 50,000 iterations. The first half of the iterations were discarded as burn-in and the remaining iterations were used to estimate the posterior distribution of the parameters. A stable potential scale reduction factor (PSFR) close to 1 was considered as evidence of chain convergence alongside inspection of trace plots and autocorrelation plots. Indirect effects were evaluated using 95% highest posterior density (HPD) credibility intervals [30]. The credibility interval indicates the probability that the parameter lies between the lower and upper bound of the interval [31]. If an interval did not include zero, the indirect effect was credible. The default non-informative prior specification in Mplus was used.

## Results

### Characteristics of cases and controls

In the matched analyses, 857 cases of severe COVID-19 and 3426 matched controls were included (for one case, only two exact matched controls were identified). Mean age was 49.9 years (*SD* 10.7) and 70.4% (*n* = 603 cases and *n* = 2 412 controls) were men. The median year that the HPA was performed was 2012 (Q1 2008, Q3 2016) for controls and 2011 (Q1 2006, Q3 2016) for cases. In the unmatched analyses, the mean age for all eligible controls was significantly lower compared to cases (43.7 years (*SD* 11.6), *p* < 0.001), and with a significantly lower proportion of men (53.8%, *p* < 0.001) compared to the matched analyses. The median year that the HPI was performed for all eligible controls was similar to the matched controls 2012 (Q1 2007, Q3 2016).

There were several differences between cases and matched controls for established COVID-19 risk factors, such as cases having higher BMI, blood pressure and presence of comorbidities as well as greater waist circumferences (Table 1). Cases also demonstrated significantly lower estVO_2_max and more unfavourable exercise patterns. There were also several differences in terms of educational level, country of birth, dietary habits and self-rated health. Moreover, cases with more severe complications from COVID-19 (death vs intensive care or hospitalization, and intensive care vs hospitalization) had significantly lower estVO_2_max, higher BMI, greater presence of comorbidities and were more often daily smokers (see Table 2).

**Table 1:** Descriptive characteristics of matched controls and cases with severe COVID-19

	***N***	**Matched controls** ***N* = 3,426**	**Cases ** ***N* = 857**	***P*-value**	**Effect size**
**EstVO**_**2**_**max**, L/min	4115	2.73 (0.48)	2.66 (0.70)	0.004	0.112
**EstVO**_**2**_**max**, ml/min/kg	4115	34.2 (5.5)	30.9 (8.1)	< 0.001	0.373
Very low, < 25 ml/min/kg		553 (16%)	166 (24%)	< 0.001	
Low, 25- < 32 ml/min/kg		1008 (30%)	251 (36%)		
Moderate, 32- < 46 ml/min/kg		1504 (44%)	243 (35%)		
High, ≥ 46 ml/min/kg		347 (10%)	28 (4.1%)		
**BMI**, kg/m^2^	4259	26.2 (2.1)	28.4 (4.6)	< 0.001	-0.516
Normal weight, < 25 kg/m^2^		1443 (42%)	186 (22%)	< 0.001	
Overweight, 25–29.9 kg/m^2^		1469 (43%)	384 (46%)		
Obesity, 30–34.9 kg/m^2^		414 (12%)	192 (23%)		
Severe obesity ≥ 35 kg/m^2^		100 (2.9%)	71 (8.5%)		
**Waist Circumference**, cm	1743	94.0 (9.1)	101.3 (12.0)	< 0.001	-0.557
≥ 88 cm (W) ≥ 102 cm (M)		504 (33%)	118 (56%)	< 0.001	
**Systolic blood pressure**, mmHg	4063	129 (9)	132 (16)	< 0.001	-0.154
≥ 140 mmHg		861 (25%)	200 (31%)	< 0.001	
**Diastolic blood pressure**, mmHg	4063	80 (6)	82 (11)	< 0.001	-0.156
≥ 90 mmHg		602 (18%)	149 (23%)	< 0.001	
**Previous chronic disease**					
Tumour	4283	269 (7.9%)	124 (14%)	< 0.001	
Diabetes	4283	72 (2.1%)	70 (8.2%)	< 0.001	
Hypertension	4283	275 (8.0%)	180 (21%)	< 0.001	
Lung disease	4283	69 (2.0%)	67 (7.8%)	< 0.001	
Cardiovascular disease	4283	254 (7.4%)	143 (17%)	< 0.001	
**Number of previous chronic diseases**	4283				
0		2775 (81%)	529 (62%)	< 0.001	
1		433 (13%)	159 (19%)		
2		161 (4.7%)	101 (12%)		
3		44 (1.3%)	50 (5.8%)		
4		13 (0.4%)	17 (2.0%)		
5		0 (0%)	1 (0.1%)		
**Exercise habits**	4205				
Never/irregular		1107 (32%)	293 (38%)	0.004	
1–2 times/week		1125 (33%)	254 (33%)		
≥ 3 times/week		1194 (35%)	232 (30%)		
**Commute type**	3149				
Passive		1568 (61%)	356 (62%)	0.297	
Low dose (< 20 min/day)		577 (22%)	131 (23%)		
High dose (≥ 20 min/day)		434 (17%)	83 (15%)		
**Physical Work Situation**	3676				
Mostly seated		1866 (61%)	328 (55%)	0.534	
Light activity		748 (24%)	164 (28%)		
Moderate/heavy activity		470 (15%)	100 (17%)		
**Diet habits**, Very poor/poor	4082	139 (4.1%)	42 (6.4%)	0.038	
**Alcohol habits**, Very poor/poor	3119	114 (4.5%)	21 (3.7%)	0.278	
**Daily smoker**	4085	294 (8.6%)	52 (7.9%)	0.750	
**Stress Overall**, Very often/often	4082	338 (9.9%)	84 (13%)	0.275	
**Perceived Health**, Very poor/poor	4085	158 (4.6%)	54 (8.2%)	0.005	
**Civil status**, Married/co-habitat	4277	1935 (57%)	480 (56%)	0.421	
**Country of birth**, Sweden	4283	3102 (91%)	686 (80%)	< 0.001	
**Educational level**	4257				
University		848 (25%)	162 (19%)	< 0.001	
High school/Voc. Education		2173 (64%)	535 (63%)		
Elementary school		383 (11%)	156 (18%)		
**Occupation group**	4049				
White collar High skilled		1.71 (55%)	396 (49%)	0.235	
White collar Low skilled		524 (16%)	169 (21%)		
Blue collar High skilled		426 (13%)	124 (15%)		
Blue collar Low skilled		515 (16%)	124 (15%)		
**Income**, thousands Swedish crowns	4283	412 (138)	397 (267)	0.121	0.053

Data presented as mean (*SD*) or n (%)

*EstVO*_*2*_*max* Estimated VO_2_max, *BMI* Body Mass Index

**Table 2:** Comparison between cases of different severity of COVID-19

	**Hospitalization ** ***N* = 547**	**Intensive care ** ***N* = 172**	**Death** ***N* = 138**	***P*-value**
**EstVO**_**2**_**max**, L/min	2.54 (0.03)	2.57 (0.05)	2.48 (0.07)	0.511
**EstVO**_**2**_**max**, ml/min/kg	31.1 (0.39)	30.7 (0.69)	28.6 (0.84)	0.032
Very low, < 25 ml/min/kg	97 (21%)	37 (27%)	32 (33%)	0.035
Low, 25- < 32 ml/min/kg	166 (36%)	45 (33%)	40 (42%)	
Moderate, 32- < 46 ml/min/kg	171 (38%)	49 (36%)	23 (24%)	
High, ≥ 46 ml/min/kg	22 (4.8%)	5 (3.7%)	1 (1.0%)	
**BMI**, kg/m^2^	27.9 (0.21)	29.2 (0.37)	29.1 (0.42)	0.002
Normal weight, < 25 kg/m^2^	129 (24%)	27 (16%)	30 (22%)	0.034
Overweight, 25–29.9 kg/m^2^	252 (48%)	69 (41%)	63 (46%)	
Obesity, 30–34.9 kg/m^2^	107 (20%)	52 (31%)	33 (24%)	
Severe obesity ≥ 35 kg/m^2^	42 (7.9%)	19 (11%)	10 (7.4%)	
**Waist Circumference**, cm	97.0 (1.00)	100.6 (1.64)	99.7 (2.26)	0.105
≥ 88 cm (W) ≥ 102 cm (M)	71 (52%)	30 (60%)	17 (65%)	0.36
**Systolic blood pressure**, mmHg	130 (0.8)	131 (1.4)	133 (1.4)	0.243
> 140 mmHg	105 (26%)	39 (31%)	56 (46%)	
**Diastolic blood pressure**, mmHg	80 (0.5)	82 (0.9)^a^	80 (1.0)	0.109
> 90 mmHg	88 (22%)	38 (30%)	23 (19%)	
**Previous chronic disease**				
Tumour	64 (12%)	20 (12%)	40 (29%)	< 0.001
Diabetes	27 (4.9%)	13 (7.6%)	30 (22%)	< 0.001
Hypertension	93 (17%)	30 (17%)	57 (41%)	< 0.001
Lung disease	36 (6.6%)	11 (6.4%)	20 (14%)	0.006
Cardiovascular disease	71 (13%)	23 (13%)	49 (36%)	< 0.001
**Number of previous chronic diseases**				
0	371 (68%)	111 (65%)	47 (34%)	< 0.001
1	94 (17%)	34 (20%)	31 (22%)	
2	54 (9.9%)	20 (12%)	27 (20%)	
3	23 (4.2%)	5 (2.9%)	22 (16%)	
4	5 (0.9%)	2 (1.2%)	10 (7.2%)	
5	0 (0%)	0 (0%)	1 (0.7%)	
**Exercise habits**				
Never/irregular	181 (36%)	67 (43%)	45 (36%)	0.71
1–2 times/week	157 (32%)	49 (31%)	48 (38%)	
≥ 3 times/week	159 (32%)	41 (26%)	32 (26%)	
**Commute type**				
Passive	212 (63%)	67 (59%)	77 (65%)	0.91
Low dose (< 20 min/day)	78 (23%)	30 (26%)	23 (19%)	
High dose (≥ 20 min/day)	47 (14%)	17 (15%)	19 (16%)	
**Physical Work Situation**				
Mostly seated	197 (55%)	65 (54%)	66 (59%)	0.94
Light activity	102 (28%)	33 (27%)	29 (26%)	
Moderate/heavy activity	61 (17%)	23 (19%)	16 (14%)	
**Diet habits**, Very poor/poor	23 (5.7%)	10 (7.5%)	9 (7.4%)	0.91
**Alcohol habits**, Very poor/poor	15 (4.5%)	4 (3.5%)	2 (1.7%)	0.71
**Daily smoker**	25 (6.2%)	4 (3.0%)	23 (19%)	< 0.001
**Stress Overall**, Very often/often	56 (14%)	17 (13%)	11 (9.1%)	0.71
**Perceived Health**, Very poor/poor	31 (7.7%)	11 (8.2%)	12 (9.9%)	0.91
**Civil status**, Married/co-habitat	308 (56%)	95 (55%)	77 (56%)	0.96
**Country of birth**, Sweden	424 (78%)	141 (82%)	121 (88%)	0.13
**Educational level**				
University	117 (22%)	28 (16%)	17 (12%)	0.23
High school/Voc. Education	335 (62%)	112 (65%)	88 (64%)	
Elementary school	91 (17%)	32 (19%)	33 (24%)	
**Occupation group**				
White collar High skilled	260 (50%)	71 (43%)	65 (49%)	0.71
White collar Low skilled	100 (19%)	36 (22%)	33 (25%)	
Blue collar High skilled	80 (16%)	24 (15%)	20 (15%)	
Blue collar Low skilled	76 (15%)	33 (20%)	15 (11%)	
**Income**, thousands Swedish crowns	380 (11)	382 (20)	356 (23)	0.62

Data presented as mean (*SE*) or n (%)

*EstVO*_*2*_*max* Estimated VO_2_max, *BMI* Body Mass Index

Mean values adjusted for sex, age and performed year

### Impact of lifestyle-related characteristics

Four models were used to quantify independent associations between potential lifestyle-related predictors and severe COVID-19 in the matched analyses, where two Model 1’s (Model 1-nc and Model 1-c) enabled comparative analyses with non-complete and complete data for all adjusting variables in Model 2 and 3, see Table 3.

**Table 3:** Odds ratio (95% CI) for lifestyle-related predictors of severe COVID-19 in matched analyses

	**Non-complete data**	**Complete data for all adjusting variables**		
	Model 1-nc	Model 1-c	Model 2	Model 3
	OR (95% CI)	OR (95% CI)	OR (95% CI)	OR (95% CI)
**EstVO**_**2**_**max,** cases/controls	689/3,426	490/3,401	490/3,401	490/3,401
per ml/min/kg	0.95 (0.94 to 0.96)	0.95 (0.94 to 0.97)	0.96 (0.95 to 0.97)	0.98 (0.97 to 0.998)
Very low, < 25 ml/min/kg	5.12 (3.30 to 7.95)	4.63 (2.80 to 7.65)	3.92 (2.36 to 6.51)	1.91 (1.09 to 3.34)
Low, 25- < 32 ml/min/kg	3.88 (2.55 to 5.91)	3.55 (2.20 to 5.72)	3.11 (1.92 to 5.02)	2.02 (1.22 to 3.35)
Moderate, 32- < 46 ml/min/kg	2.35 (1.55 to 3.55)	2.29 (1.43 to 3.65)	2.08 (1.30 to 3.33)	1.62 (1.004 to 2.62)
High, ≥ 46 ml/min/kg	1 (ref)	1 (ref)	1 (ref)	1 (ref)
**BMI,** cases/controls	833/3,426	490/3,401	490/3,401	490/3,401
per unit kg/m^2^	1.13 (1.11 to 1.15)	1.12 (1.10 to 1.14)	1.11 (1.09 to 1.14)	1.09 (1.06 to 1.12)
Normal weight, < 25 kg/m^2^	1 (ref)	1 (ref)	1 (ref)	1 (ref)
Overweight, 25–29.9 kg/m^2^	2.11 (1.74 to 2.56)	2.32 (1.82 to 2.97)	2.23 (1.75 to 2.86)	1.98 (1.53 to 2.56)
Obesity, 30–34.9 kg/m^2^	3.81 (3.02 to 4.82)	4.12 (3.07 to 5.52)	3.77 (2.80 to 5.08)	2.94 (2.13 to 4.07)
Severe obesity ≥ 35 kg/m^2^	5.93 (4.20 to 8.36)	4.81 (3.05 to 7.58)	4.39 (2.78 to 6.95)	2.98 (1.80 to 4.94)
**Waist Circumference,** cases/controls	212/1,531	157/1,520	157/1,520	157/1,520
per cm	1.05 (1.04 to 1.07)	1.05 (1.03 to 1.06)	1.05 (1.03 to 1.06)	1.04 (1.02 to 1.06)^a^
< 88 cm (W) or < 102 cm (M)	1 (ref)	1 (ref)	1 (ref)	1 (ref)^a^
≥ 88 cm (W) ≥ 102 cm (M)	2.58 (1.92 to 3.49)	2.42 (1.73 to 3.39)	2.34 (1.67 to 3.29)	1.75 (1.20 to 2.55)
**Systolic blood pressure,** cases/controls	646/3,417	490/3,392	490/3,392	490/3,392
per mmHg	1.01 (1.006 to 1.02)	1.01 (1.002 to 1.02)	1.01 (1.002 to 1.02)	1.00 (0.99 to 1.01)
< 140 mmHg	1 (ref)	1 (ref)	1 (ref)	1 (ref)
≥ 140 mmHg	1.35 (1.11 to 1.64)	1.29 (1.03 to 1.62)	1.27 (1.01 to 1.59)	0.95 (0.74 to 1.20)
**Diastolic blood pressure,** cases/controls	646/3,417	490/3,392	490/3,392	490/3,392
per mmHg	1.02 (1.01 to 1.03)	1.02 (1.01 to 1.03)	1.02 (1.01 to 1.03)	1.01 (0.997 to 1.02)
< 90 mmHg	1 (ref)	1 (ref)	1 (ref)	1 (ref)
≥ 90 mmHg	1.51 (1.22 to 1.87)	1.51 (1.19 to 1.93)	1.54 (1.20 to 1.96)	1.16 (0.90 to 1.50)
**Number of chronic diseases,** cases/controls	857/3,426	490/3,401	490/3,401	490/3,401
0	1 (ref)	1 (ref)	1 (ref)	1 (ref)
1	2.04 (1.65 to 2.52)	1.99 (1.53 to 2.57)	2.00 (1.54 to 2.61)	1.88 (1.44 to 2.45)
2	3.66 (2.76 to 4.84)	2.76 (1.92 to 3.95)	2.75 (1.91 to 3.96)	2.38 (1.64 to 3.45)
3	6.64 (4.32 to 10.21)	4.35 (2.54 to 7.44)	4.45 (2.58 to 7.67)	3.86 (2.22 to 6.71)
4 to 5	8.09 (3.90 to 16.82)	5.15 (2.18 to 12.20)	5.63 (2.37 to 13.35)	4.55 (1.83 to 11.33)
**Exercise habits,** cases/controls	779/3,426	490/3,401	490/3,401	490/3,401
Never/irregular	1 (ref)	1 (ref)	1 (ref)	1 (ref)
1–2 times/week	0.85 (0.70 to 1.02)	0.95 (0.76 to 1.20)	1.01 (0.80 to 1.28)	1.14 (0.89 to 1.46)
≥ 3 times/week	0.75 (0.62 to 0.91)	0.87 (0.69 to 1.11)	0.92 (0.72 to 1.17)	1.14 (0.88 to 1.48)
**Commute type,** cases/controls	570/2,579	425/2,561	425/2,561	425/2,561
Passive	1 (ref)	1 (ref)	1 (ref)	1 (ref)
Low dose (< 20 min/day)	1.01 (.80 to 1.26)	1.04 (0.81 to 1.34)	1.08 (0.84 to 1.40)	1.17 (0.90 to 1.52)
High dose (≥ 20 min/day)	0.88 (0.67 to 1.15)	0.92 (0.69 to 1.25)	0.96 (0.71 to 1.31)	1.11 (0.81 to 1.52)
**Physical Work Situation,** cases/controls	592/3,084	426/3,049	426/3,049	426/3,049
Mostly seated	1 (ref)	1 (ref)	1 (ref)	1 (ref)
Light activity	1.22 (0.99 to 1.50)	1.22 (0.97 to 1.55)	1.04 (0.81 to 1.33)	1.01 (0.79 to 1.30)
Moderate/heavy activity	1.19 (0.93 to 1.53)	1.17 (0.88 to 1.55)	0.99 (0.74 to 1.33)	1.03 (0.76 to 1.39)
**Diet habits,** cases/controls	659/3,423	490/3,400	490/3,400	490/3,400
Neutral/Good/Very good	1 (ref)	1 (ref)	1 (ref)	1 (ref)
Very poor/poor	1.40 (0.97 to 2.03)	1.47 (0.98 to 2.19)	1.46 (0.97 to 2.19)	1.24 (0.81 to 1.89)
**Alcohol habits,** cases/controls	567/2,552	423/2,536	423/2,536	423/2,536
Neutral/Good/Very good	1 (ref)	1 (ref)	1 (ref)	1 (ref)
Very poor/poor	0.73 (0.45 to 1.18)	0.61 (0.34 to 1.10)	0.68 (0.38 to 1.23)	0.65 (0.36 to 1.20)
**Daily smoker,** cases/controls	659/3,426	490/3,401	490/3,401	490/3,401
Never smoker/Occasionally	1 (ref)	1 (ref)	1 (ref)	1 (ref)
Daily smoker	0.79 (0.58 to 1.08)	0.75 (0.52 to 1.08)	0.64 (0.44 to 0.93)	0.60 (0.41 to 0.89)
**Stress Overall,** cases/controls	659/3,423	490/3,401	490/3,401	490/3,401
Sometimes/rarely/never	1 (ref)	1 (ref)	1 (ref)	1 (ref)
Very often/often	1.30 (1.002 to 1.70)	1.39 (1.04 to 1.86)	1.42 (1.06 to 1.91	1.36 (1.001 to 1.84)

*EstVO*_*2*_*max* Estimated VO_2_max, *BMI* Body Mass Index

Model 1; adjusted for sex, age and performed year

Model 2; additionally adjusted for educational level, civil status, and country of birth

Model 3: additionally adjusted for estimated VO_2_max, BMI, number of chronic diseases, exercise habits, smoking, overall stress

^a^ adjusted as model 3, except for BMI

In terms of CRF, there was a graded increase in odds with lower compared to high levels, OR = 1.62 (95% CI, 1.00 to 2.62) for moderate CRF (32 to < 46 ml·min^−1^·kg^−1^) and an approximately two-fold increased odds for low (OR = 2.02, 1.22 to 3.35) and very low fitness (OR = 1.91, 1.09 to 3.34), respectively (Table 3). Similarly, being overweight was associated with two-fold increased odds compared to normal weight (OR = 1.98, 1.53 to 2.56), and obesity and severe obesity was associated with three-fold increased odds (OR = 2.94, 2.13 to 4.07 and OR = 2.98, 1.80 to 4.94 respectively). A larger WC was associated with higher odds in the fully adjusted model, OR = 1.75, 1.20 to 2.55. Presence of chronic disease had a graded increase for every additional diagnosis, OR = 1.88 (95% CI: 1.44 to 2.45) for one chronic disease, and OR = 4.55 (1.83 to 11.33) for 4 to 5 chronic diseases. Neither high systolic nor diastolic blood pressure remained significantly associated with severe COVID-19 after multi-adjustment. Reporting daily smoking (OR = 0.60, 0.41 to 0.89) as well as a high level of stress (OR = 1.36, 1.001 to 1.84) were significantly associated with severe COVID-19 in the fully adjusted model.

**Fig. 1:**
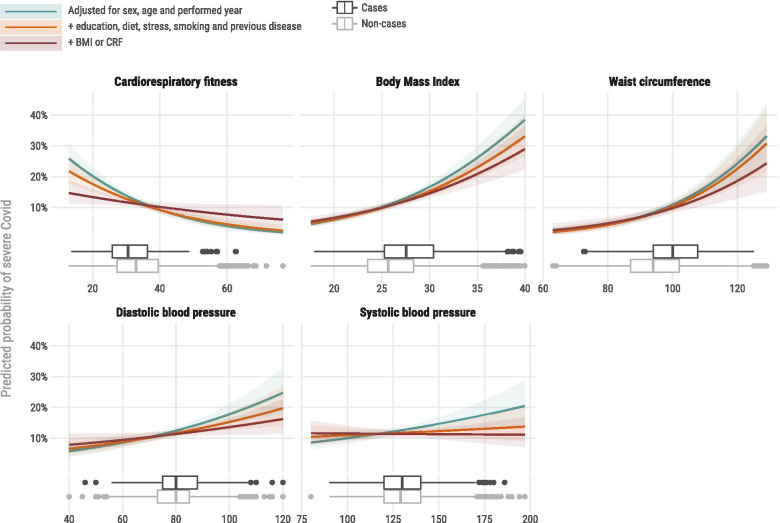
Marginal effects plot for severe COVID-19 (lines) and box-plot graphs for distribution (below lines). Probability in the figures describes the point estimate of predicted probability for severe COVID-19 at conventional risk cut-off for each variable and how that probability change with increased adjustment for confounders

Figure 1 presents the predicted probability of severe COVID-19 according to continuous levels of CRF, overall and central obesity (BMI and WC), and systolic and diastolic blood pressure. All obesity and blood pressure measures were attenuated by adjustment for lifestyle variables and CRF, however, these associations with severe COVID-19 remained significant.

### Impact of sociodemographic factors

Low education (elementary vs. university) predicted severe COVID-19 (OR = 1.81, 1.28 to 2.54) after multi-adjustment, as did being born outside Sweden vs. being born in Sweden (OR = 2.58, 1.97 to 3.38) (Table 4). No significant associations were seen for civil status, occupational groups, or income quartiles.

**Table 4:** Odds ratio (95% CI) for sociodemographic predictors of severe COVID-19 in matched analyses

	**Non-complete data**	**Complete data for all adjusting variables**		
	Model 1	Model 1	Model 2	Model 3
	OR (95% CI)	OR (95% CI)	OR (95% CI)	OR (95% CI)
**Educational level**, cases/controls	853/3,404	490/3,401	490/3,401	490/3,401
University	1 (ref)	1 (ref)	1 (ref)	1 (ref)
High school/Voc. Education	1.29 (1.06 to 1.56)	1.32 (1.03 to 1.69)	1.37 (1.06 to 1.76)	1.20 (0.92 to 1.56)
Elementary school	2.07 (1.60 to 2.68)	2.10 (1.51 to 2.90)	2.07 (1.49 to 2.88)	1.81 (1.28 to 2.54)
**Civil status**, cases/controls	856/3,421	490/3,401	490/3,401	490/3,401
Married/co-habitat	1 (ref)	1 (ref)	1 (ref)	1 (ref)
Single/divorced/widower	0.97 (0.83 to 1.13)	0.99 (0.81 to 1.21)	1.01 (0.83 to 1.24)	1.03 (0.84 to 1.27)
**Country of birth**, cases/controls	857/3,426	490/3,401	490/3,401	490/3,401
Sweden	1 (ref)	1 (ref)	1 (ref)	1 (ref)
Else	2.50 (2.04 to 3.07)	2.57 (1.98 to 3.33)	2.57 (1.98 to 3.34)	2.58 (1.97 to 3.38)
**Occupation group**, cases/controls	813/3,236	471/3,224	471/3,224	471/3,224
White collar High skilled	1 (ref)	1 (ref)	1 (ref)	1 (ref)
White collar Low skilled	1.44 (1.16 to 1.78)	1.43 (1.09 to 1.88)	1.16 (0.87 to 1.54)	1.04 (0.77 to 1.39)
Blue collar High skilled	1.30 (1.03 to 1.64)	1.31 (0.96 to 1.77)	0.99 (0.72 to 1.37)	0.95 (0.68 to 1.32)
Blue collar Low skilled	1.07 (0.86 to 1.34)	1.24 (0.94 to 1.64)	0.86 (0.63 to 1.18)	0.81 (0.59 to 1.12)
**Income, thousands Swedish crowns**, cases/controls	857/3,426	490/3,401	490/3,401	490/3,401
Q4	1 (ref)	1 (ref)	1 (ref)	1 (ref)
Q3-Q2	0.97 (0.80 to 1.18)	1.11 (0.85 to 1.45)	0.89 (0.67 to 1.17)	0.82 (0.60 to 1.23)
Q1	1.32 (1.04 to 1.68)	1.45 (1.05 to 1.99)	1.00 (0.71 to 1.42)	0.82 (0.62 to 1.09)

Model 1; adjusted for sex, age and performed year

Model 2; additionally adjusted for civil status and country of birth

Model 3: additionally adjusted for estimated VO_2_max, BMI, number of chronic diseases, exercise habits, smoking, overall stress

In additional sensitivity analyses using unmatched controls (see Additional file 3, Supplement Tables 1 and 2) the odds for severe COVID-19 were higher in men (Model 3, OR = 1.97, 1.62 to 2.40) and with increasing age (Model 3, per year OR = 1.02, 1.01 to 1.03). Individuals ≥ 70 years and 60 to 69 years had higher odds compared to those < 60 years. However, the odds were attenuated by additional adjustments for lifestyle-related factors (Model 2 and 3 adjustment). Further, lifestyle-related and sociodemographic predictors showed similar associations as in the matched analyses, with only marginal variations in both OR and CI, which did not alter the results or conclusions of the unmatched analyses.

### Mediation analyses

**Fig. 2:**
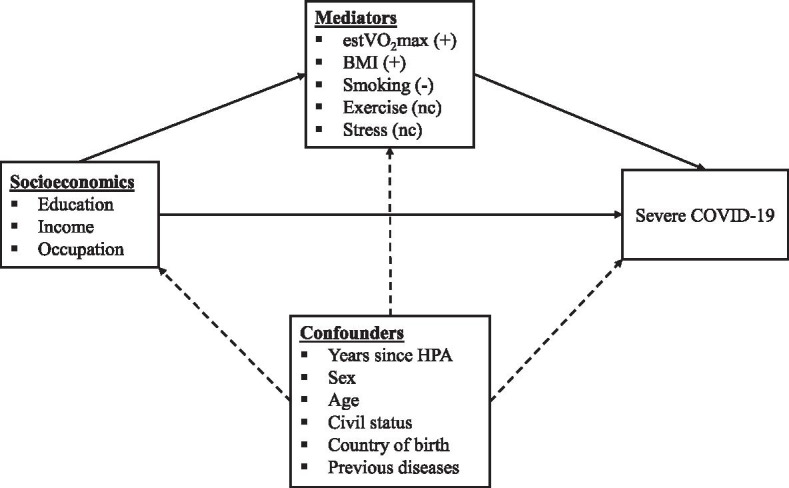
Mediation model showing indirect effects of socioeconomic factors on severe COVID-19 in matched analyses and included confounders. ( +) indicates a positive indirect effect, (-) indicates a negative indirect effect, and (nc) indicates no credible indirect effect

The mediation analyses are summarized in Table 5 and Fig. 2. Indirect effects were observed through BMI, CRF and smoking, whereas no credible indirect effects were observed through exercise and stress. The proportion mediated ranged from 12 to 23% for BMI, 9% to 17% for CRF, and 24% to 54% for smoking. Compared to those in the highest socioeconomic category, lower socioeconomic status was related to an increased risk of severe COVID-19 through BMI and CRF, and a lower risk for severe COVID-19 through smoking. The findings were similar across all three socioeconomic indicators. The total proportion mediated across all five mediators ranged from 49 to 86%. Because the mediation models were inconsistent (i.e., they included both positive and negative effects on the dependent variable), we calculated the proportion mediated based on absolute values [29]. Thus, these values represent the proportion of the absolute total effect that was mediated. Mediation analyses using an unmatched sample (*N* = 279,455) showed similar results (see Additional file 3, Supplement Table 3).

**Table 5:** Indirect effects of socioeconomic factors on severe COVID-19 in matched analyses

	**BMI**	**CRF**	**Smoking**^**a**^	**Exercise**	**Stress**	**Total proportion mediated**
	*ab* (95% HPD CI)	*ab* (95% HPD CI)	*ab* (95% HPD CI)	*ab* (95% HPD CI)	*ab* (95% HPD CI)	
**Educational level**						
High (0) vs low (1)	0.090 (0.060 to 0.122)	0.052 (0.019 to 0.087)	-0.175 (-0.317 to -0.049)	0.001 (-0.010 to 0.013)	-0.004 (-0.015 to 0.003)	
Proportion mediated	0.157	0.090	0.304	0.002	0.007	0.560
High (0) vs medium (1)	0.069 (0.047 to 0.092)	0.034 (0.012 to 0.057)	-0.135 (-0.246 to -0.038)	0.001 (-0.008 to 0.010)	-0.004 (-0.015 to 0.003)	
Proportion mediated	0.230	0.113	0.450	0.003	0.013	0.810
**Income**						
Q4 (0) vs Q1 (1)	0.051 (0.027 to 0.077)	0.049 (0.020 to 0.079)	-0.127 (-0.241 to -0.027)	0.002 (-0.007 to 0.011)	-0.003 (-0.012 to 0.004)	
Proportion mediated	0.172	0.166	0.429	0.007	0.010	0.784
Q4 (0) vs Q2-Q3 (1)	0.035 (0.017 to 0.054)	0.027 (0.011 to 0.045)	-0.079 (-0.160 to -0.013)	0.002 (-0.007 to 0.011)	-0.003 (-0.010 to 0.003)	
Proportion mediated	0.117	0.091	0.265	0.007	0.010	0.490
**Occupation group**						
WCHS (0) vs BCLS (1)	0.056 (0.034 to 0.080)	0.034 (0.013 to 0.057)	-0.148 (-0.273 to -0.041)	0.002 (-0.010 to 0.014)	-0.004 (-0.015 to 0.005)	
Proportion mediated	0.159	0.097	0.420	0.006	0.011	0.693
WCHS (0) vs BCHS (1)	0.048 (0.026 to 0.073)	0.030 (0.011 to 0.052)	-0.145 (-0.266 to -0.036)	0.003 (-0.014 to 0.020)	-0.006 (-0.019 to 0.006)	
Proportion mediated	0.178	0.111	0.537	0.011	0.022	0.859
WCHS (0) vs WCLS (1)	0.058 (0.035 to 0.083)	0.028 (0.010 to 0.049)	-0.100 (-0.192 to -0.022)	0.000 (-0.004 to 0.003)	-0.002 (-0.010 to 0.003)	
Proportion mediated	0.222	0.107	0.383	0.000	0.008	0.720

Adjusted for sex, age, year HPA was performed, civil status, country of birth, and number of previous diseases as confounders of the exposure-mediator, exposure-outcome, and mediator-outcome relation

^a^Smoking was coded as a binary variable (0 = never/seldom, 1 = daily smoker)

*ab *Indirect effect, *HPD CI* Highest posterior density credibility interval, *BMI* Body mass index, *CRF* Cardiorespiratory fitness, *Q* Quartile, *WCHS* White-collar high-skilled, *WCLS* White-collar low-skilled, *BCLS* Blue-collar low-skilled, *BCHS* Blue-collar high-skilled

## Discussion

The main results of the present study include strong associations of several lifestyle-related risk factors, including CRF, overweight/obesity, perceived stress, and high blood pressure, with severe COVID-19, even after adjustments for sociodemographic factors and previous diseases. Among patients with severe COVID-19, those with more severe COVID-19 (death vs intensive care or hospitalization, and intensive care vs hospitalization) had lower CRF. In mutually adjusted analyses, higher CRF attenuated some of the risks related to both obesity and hypertension. Low educational level, low income as well as blue collar/low skilled occupations were associated with increased risk of severe COVID-19. However, these associations were, to a relatively large proportion, mediated by CRF, BMI and smoking. The results were consistent when using matched or unmatched controls.

### Comparison with other studies

This is, to our knowledge, the first study investigating the association between a wide variation of lifestyle-related risk factors, including CRF, and severe COVID-19. The results are consistent with the few previous existing studies that have found evidence of associations between PA, overweight/obesity and smoking with severe COVID-19 [9, 10]. In 387,109 middle-aged men and women from the UK Biobank, there were 760 cases of hospitalization for COVID-19 [9]. After multi-adjustment, participants reporting no regular PA had a 32% higher risk of hospitalization for COVID-19 compared to those reporting some PA (active but below guidelines) or meeting activity guidelines (≥ 150 min per week of moderate PA or 75 min per week of vigorous PA). Smoking, compared to not smoking, was associated with a 42% higher risk. Further, a lifestyle score was derived including both physical inactivity, smoking, heavy alcohol consumption, and overweight/obesity, which showed a dose-dependent increased risk of hospitalization for COVID-19 partly explained by C-reactive protein levels. Moreover, in 48,440 adult patients with a COVID-19 diagnosis, those who had been consistently inactive (0–10 min of PA per week) in the two years preceding COVID-19 infection, had a significantly higher odds of hospital admission (OR 2.26), admission to intensive care (OR 1.73) and death (OR 2.49) due to COVID-19 compared to patients reporting being inconsistently (10 to < 150 min per week) or consistently (≥ 150 min per week) moderately to vigorously physically active [10]. Although the present study did not find a significant association between PA levels and severe COVID-19, the strong and consistent association of CRF and COVID-19 may be even more important. All previous studies have relied on self-reported PA, which is a subjective measure of recent PA levels containing well-known errors (recall-bias) that permit valid analyses on mainly aggregated PA levels [32]. In the present analyses, CRF was included as a more objective measure of recent PA as well as an indicator of the status of the cardiorespiratory system. This showed a lower risk of severe COVID-19 per ml·min^−1^·kg^−1^ with a doubling of risk between the two lowest and the highest categories (< 32 ml·min^−1^·kg^−1^ and ≥ 46 ml·min^−1^·kg^−1^ respectively). This is similar to a previous report on all-cause mortality and CVD morbidity risk, where decreases of 2.3% and 2.6% per ml increase in estVO_2_max were seen [33]. Only one previous study has studied the association between recent CRF and COVID-19. In a small sample of patients (*n* = 246) with positive tests for COVID-19, men (but not women) with lower CRF were more likely to be hospitalized than those with higher CRF [11]. A study using data from military conscript (≈18 years of age) between 1968 and 2005 showed that high CRF at conscript was associated with lower odds of severe COVID-19 later in life [34].

In the fully adjusted analyses, both perceived stress and smoking remained significantly associated with severe COVID-19. Reporting high overall stress was associated with significantly higher OR (1.36) compared to low stress. This is partly supported by findings from the UK Biobank participants [17] where a 58% increased risk of hospitalization due to COVID-19 was found among individuals reporting high psychological distress. In contrast to the present results, the association did not remain after full adjustment with comorbidities, other lifestyle variables and socioeconomics. More surprisingly in the present study, smokers had a significantly lower OR (0.60) compared to non-smokers, which adds to equivocal results in the current literature [4, 9]. A hypothesis has been raised that nicotine may have beneficial effects on COVID-19 due to its interaction with the renin-angiotensin and effects on the immunomodulatory system [35], but further investigation of the mechanisms associated with these findings remains to be elucidated by better controlled studies.

Consistent with other publications [5, 6], both overweight and obesity were associated with a higher risk of severe COVID-19. This could partly be explained by a higher prevalence of metabolic risk factors and low-grade inflammation in overweight/obese individuals, as these have been identified as central mechanisms for a higher vulnerability to severe COVID-19 [36]. Interestingly, a recent paper including over 17 million individuals found similar associations between commonly accepted risk factors (age, male sex, deprivation, obesity, and some comorbidities) for non-COVID (including CVD, cancer, dementia etc.) deaths and for COVID-19 deaths, suggesting that COVID-19 largely mirrors existing risks faced by patients [37]. However in the present study, obesity-risk was at least partly attenuated by CRF. Attenuation by CRF were also seen for central obesity (waist circumference) and high systolic and diastolic blood pressure related risks. These findings are highly clinically relevant and in line with previous studies on cardiovascular disease risk and premature death, where “fat but fit” individuals had significantly better prognoses for cardiovascular outcomes and mortality compared to obese but unfit individuals [38, 39].

There are several suggested mechanisms for the beneficial effects of regular PA and higher CRF levels on both COVID-19 severity per se, as well as attenuation of the obesity- and hypertension-related risks [12, 13, 16, 40]. One is the lower prevalence of obesity and hypertension in more active individuals [6, 15]. Moreover, regular exercise induces a marked increase in several anti-inflammatory cytokines, counteracting the low-grade inflammatory state present in many chronic metabolic diseases (such as obesity and type 2 diabetes) [12, 13]. It also induces a natural immune-protection against more severe COVID-19 by reducing the so-called “cytokine storm” (peaking of pro-inflammatory cytokines including interleukin-6 and tumour necrosis factor-alpha) that ICU-patients with severe COVID-19 experience [41, 42]. Also, regular PA has shown a direct and positive effect on lung function, and the antibody concentration after vaccination is higher among regularly physically active individuals [40].

There were differences in sociodemographic factors between cases and controls in the present study, which is consistent with previous studies. Among 431,051 British adults, low levels of education, income and area deprivation doubled the risk of hospitalization due to COVID-19, with a 39% higher risk for those with occupations including personal service and sales compared to managers [17]. Across 3135 US counties, the counties with a higher percentage of households with poor housing had a higher incidence of COVID-19, as well as mortality due to COVID-19 [18]. These findings are supported by a large Swedish study, indicating that an educational level only up to elementary school, compared to higher educational levels, was associated with a higher risk for both intensive care and non-intensive care hospitalisation due to COVID-19 [2]. Also, blue-collar workers were significantly less likely to work from home or to change commuting habits in relation to the COVID-19 pandemic, compared to white-collar workers [43]. However, as health status prior to infection seems to heavily impact the severity of COVID-19, we hypothesized that the variation in health lifestyle factors would mediate some of the risk associated with socioeconomic factors. In the mediation analyses, lower socioeconomic status (indicated by education, income, and occupation) was related to an increased risk of severe COVID-19 through higher BMI and lower CRF, whereas lower socioeconomic status was related to a lower risk of severe COVID-19 through smoking. Similar mediation analyses have been performed for cardiovascular disease [19] and cancer morbidity and mortality [20], where modifiable factors including BMI and smoking explained between 42 and 46% of the association between low socioeconomic position and the outcomes. The proportion mediated in the current study ranged from 49 to 86%, indicating that the mediators accounted for a relatively large proportion of the association between socioeconomic factors and risk of severe COVID-19. The present indirect effects on severe COVID-19 risk through BMI and CRF highlight factors that could be targeted in interventions to strengthen the resilience for future severe infections.

### Strengths and weaknesses of the study

A case–control study is not as powerful as other types of studies in confirming a causal relationship [44]. However, the strengths of this study are the large cohort of different aged women and men with variations in socioeconomic gradients, and the available data on several lifestyle-related factors assessed by standardised methods. Another strength is the highly corresponding results obtained using either the sex- and age-matched controls or all eligible controls in the analyses. The mediation analyses are also a strength, as they highlight processes through which socioeconomic inequalities may influence disease risk. In Sweden, patients from both low and high socioeconomic status have similar access to healthcare, which strengthens the argument for the role of lifestyle factors, including CRF, in preventing severe COVID-19. Limitations of the study include self-reported data regarding lifestyle habits, which risks recall bias [45]. However, questionnaires with categorical answer modes as used in the present study have been reported to provide superior validity compared to open answer modes for PA level [46]. The study design explores associations over time, but does not give information about causality, in this case between lifestyle related and socioeconomic risk factors and severe COVID-19. Moreover, the clinical status of the cases and controls between the time of their HPA and the follow-up period were not monitored. There is a risk of reversed causality due to individuals with a better health status possibly having higher CRF, lower BMI and lower blood pressure. However, the size of the study population made it possible to identify the effect of low CRF, obesity and elevated blood pressure by adjusting for multiple potential confounders and thereby reducing the risk of reverse causality.

## Conclusions

Higher CRF was associated with better resilience for severe COVID-19, which is of great clinical value, particularly for high-risk individuals with obesity and/or hypertension. Further, the mediation analyses included in the present paper add important initial evidence of modifiable factors mediating the associations between socioeconomic variables and severe COVID-19. This should shift the focus from structural factors, such as educational level or income per se, having direct effects on disease risk, to instead highlighting and targeting modifiable factors, including CRF and BMI, to increase resilience. This is particularly important as a decrease by 10% (4.2 ml·min^−1^·kg^−1^) in CRF has been reported over the last two decades in the Swedish working population [47]. This has been confirmed in international data [48]. During the same time period, the prevalence of obesity and severe obesity has increased by 153% and 86%, respectively [49]. This calls for an urgent need to implement interventions, such as PA on prescription, to increase CRF, preferably specifically targeting high-risk individuals. Further analyses on how sex and age moderate the association between CRF and severe COVID-19 are needed, as are studies including objective measures (e.g. accelerometers) for assessment of PA patterns. Although the mediation analyses highlight processes through which socioeconomic inequalities may influence disease risk, given the correlational nature of the data, these findings need to be replicated in future studies using designs that allow for stronger causal conclusions.

## Supplementary Information

**Additional file 1:** **Supplement figure 1.** Flow chart of included and excluded participants. Contains a flow chart of included and excluded cases and controls.

**Additional file 2.** Questions from Health Profile Assessment. Contains the questions from the Health Profile Assessment for self-reported data in the study.

**Additional file 3. **Supplement Tables. Contains supplement table 1 to 3.

## Data Availability

The datasets generated and/or analysed during the current study are not publicly available due to them being the property of the HPI Health Profile Institute, but are available from the corresponding author, eline@gih.se. Additional information regarding technical details, statistical code, and derived data are also available from the corresponding author.

## Abbreviations

BCHS: Blue-collar high-skilled.
BCLS: Blue-collar low-skilled,
BMI: Body mass index
BP: Blood pressure
CRF: Cardiorespiratory fitness
COVID-19: Coronavirus disease 2019
estVO2max: Estimated maximal oxygen consumption
HPA: Health Profile Assessment
HPD CI: Highest posterior density credibility interval,
ICD: International Classification of Diseases
OR: Odds ratio
PA: Physical activity
Q: Quartile
VO2max: Maximal oxygen consumption
WCHS: White-collar high-skilled
WCLS: White-collar low-skilled
