# Supplementary material for: Cardiorespiratory fitness and lifestyle on severe COVID-19 risk in 279,455 adults: a case control study
Source: Int J Behav Nutr Phys Act. 2021 Oct 19;18:135. doi: 10.1186/s12966-021-01198-5 (PMC8524225; doi:10.1186/s12966-021-01198-5)
Supplement: Supplementary file 1 — Additional file 1: Supplement figure 1. Flow chart of included and excluded participants. Contains a flow chart of included and excluded cases and controls. [file 12966_2021_1198_MOESM1_ESM.pdf]

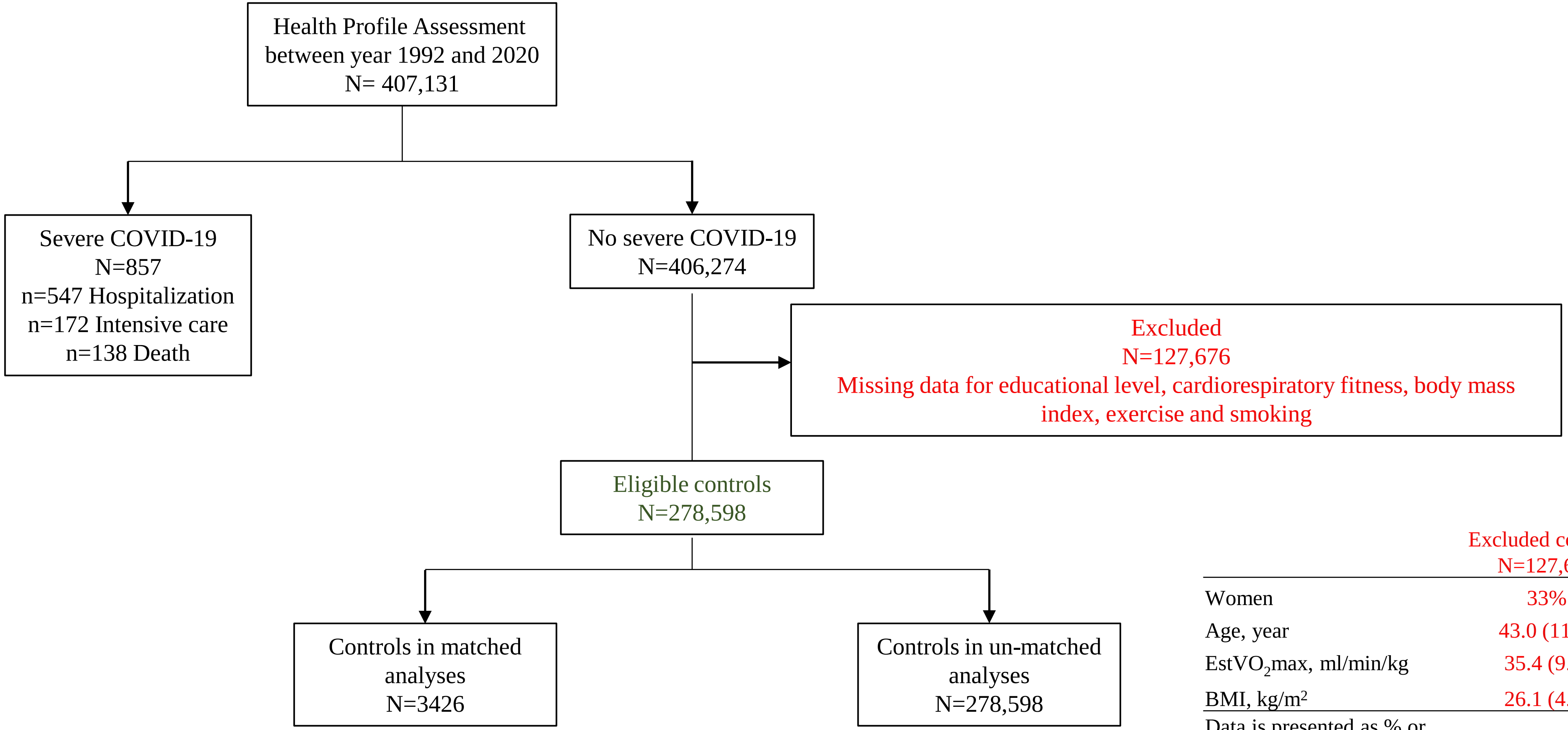

	Excluded controls N=127,676	Eligable controls N=278,598	Differences[a]
Women	33%	46%	$p<0.001$
Age, year	43.0 (11.9)	43.7 (11.6)	$p<0.001$
$EstVO_2max$, ml/min/kg	35.4 (9.8)	36.1 (10.0)	$p<0.001$
BMI, kg/m^2	26.1 (4.2)	25.7 (4.1)	$p<0.001$

Data is presented as % or mean (SD)

[a] Potential differences between included and excluded participants were assessed using independent t-test (continuous data) and chi2-test (categorical data)

Supplement figure 1. Flowchart of included and excluded participants, with a comparison of central variables
