# Supplementary material for: Cardiorespiratory fitness and lifestyle on severe COVID-19 risk in 279,455 adults: a case control study
Source: Int J Behav Nutr Phys Act. 2021 Oct 19;18:135. doi: 10.1186/s12966-021-01198-5 (PMC8524225; doi:10.1186/s12966-021-01198-5)
Supplement: Supplementary file 2 — Additional file 2. Questions from Health Profile Assessment. Contains the questions from the Health Profile Assessment for self-reported data in the study. [file 12966_2021_1198_MOESM2_ESM.docx]

**Questions from Health Profile Assessment**

**Exercise habits**

*I exercise for the purpose of maintaining/improving my physical fitness, health and well-being...*

□ Never
□ Sometimes
□ 1-2 times/week
□ 3-5 times/week 6 times/week
□ At least 6 times/week

**Commute type**

*I walk or cycle to and/or from work…*

□ Less than 5 minutes/day
□ 5-9 minutes/day
□ 10-19 minutes/day
□ 20-29 minutes/day
□ At least 30 minutes/day

**My physical work situation**

*I consider my physical work situation as…*

□ Sitting with some movement,
□ Physically active
□ Occasionally physically demanding
□ Occasionally very physically demanding

**Diet habits**

*I consider my diet, regarding both meal frequency and nutritional content to be ...*

□ Very poor
□ Poor
□ Neither good or bad
□ Good
□ Very good

**Alcohol habits**

*I consider my alcohol habits, from a health perspective to be ...*

□ Very poor
□ Poor
□ Neither good or bad
□ Good
□ Very good

**Smoking habits**
*I smoke...*

□ At least 20 cig/day
□ 11-19 cig/day
□ 1-10 cig/day
□ Occasionally
□ Never

**Overall stress**
*I perceive stress in my life, both personally and at work...*

□ Very often
□ Often
□ Sometime
□ Rarely
□ Never

**Perceived symptoms of anxiety and depression**

*I experience worry, sadness or anxiety...*

□ Very often
□ Often
□ Sometime
□ Rarely
□ Never

**Perceived health**

*I perceive my physical and mental health as...*
□ Very poor
□ Poor
□ Neither good or bad
□ Good
□ Very good
