# Supplementary material for: Cardiorespiratory fitness and lifestyle on severe COVID-19 risk in 279,455 adults: a case control study
Source: Int J Behav Nutr Phys Act. 2021 Oct 19;18:135. doi: 10.1186/s12966-021-01198-5 (PMC8524225; doi:10.1186/s12966-021-01198-5)
Supplement: Supplementary file 3 — Additional file 3. Supplement Tables. Contains supplement table 1 to 3. [file 12966_2021_1198_MOESM3_ESM.docx]

**Supplement Tables**

| **Supplement Table 1.** Odds ratio (95% CI) for lifestyle-related predictors of severe COVID-19 in unmatched analyses | | | | | | |  |
| --- | --- | --- | --- | --- | --- | --- | --- |
|  | **Non-complete data** | | **Complete data for all adjusting variables** | | | |  |
|  | Model 1-nc | | Model 1-c | Model 2 | | Model 3 |  |
|  | OR (95% CI) | | OR (95% CI) | OR (95% CI) | | OR (95% CI) |  |
| **Sex** | 857/278,598 | | 490/276,818 | 490/276,818 | | 490/276,818 |  |
| Women | 1 (ref) | | 1 (ref) | 1 (ref) | | 1 (ref) |  |
| Men | 2.19 (1.89 to 1.54) | | 2.15 (1.77 to 2.60) | 2.08 (1.72 to 2.52) | | 1.97 (1.62 to 2.40) |  |
|  |  | |  |  | |  |  |
| **Age** | 857/278,598 | | 490/276,818 | 490/276,818 | | 490/276,818 |  |
| per year | 1.05 (1.04 to 1.05) | | 1.04 (1.03 to 1.05) | 1.04 (1.03 to 1.05) | | 1.02 (1.01 to 1.03) |  |
| <60 | 1 (ref) | | 1 (ref) | 1 (ref) | | 1 (ref) |  |
| 60-69 | 2.34 (1.98 to 2.77) | | 2.01 (1.59 to 2.53) | 1.78 (1.41 to 2.28) | | 1.30 (1.01 to 1.68) |  |
| >=70 | 9.42 (4.17 to 21.3) | | 6.63 (1.64 to 26.8) | 5.65 (1.39 to 23.0) | | 3.57 (0.87 to 14.7) |  |
|  |  | |  |  | |  |  |
| **EstVO2max**, cases/controls | 689/278,598 | | 490/276,818 | 490/276,818 | | 490/276,818 |  |
| per ml | 0.95 (0.94 to 0.96) | | 0.95 (0.94 to 0.96) | 0.96 (0.95 to 0.97) | | 0.98 (0.97 to 0.993) |  |
| <25 ml/min/kg | 5.84 (3.85 to 8.85) | | 5.11 (3.17 to 8.23) | 4.43 (2.74 to 7.15) | | 2.28 (1.35 to 3.84) |  |
| 25-<32 ml/min/kg | 4.33 (2.90 to 6.46) | | 3.91 (2.48 to 6.17) | 3.49 (2.21 to 5.53) | | 2.44 (1.52 to 3.93) |  |
| 32-<46 ml/min/kg | 2.56 (1.72 to 3.80) | | 2.39 (1.53 to 3.74) | 2.25 (1.44 to 3.52) | | 1.91 (1.21 to 3.00) |  |
| ≥46 ml/min/kg | 1 (ref) | | 1 (ref) | 1 (ref) | | 1 (ref) |  |
|  |  | |  |  | |  |  |
| **BMI, per unit**, cases/controls | 833/278,598 | | 490/276,818 | 490/276,818 | | 490/276,818 |  |
| per kg/m^2^ | 1.12 (1.11 to 1.14) | | 1.11 (1.10 to 1.13) | 1.11 (1.09 to 1.13) | | 1.08 (1.06 to 1.10) |  |
| Normal weight, <25 kg/m^2^ | 1 (ref) | | 1 (ref) | 1 (ref) | | 1 (ref) |  |
| Overweight, 25-29.9 kg/m^2^ | 2.10 (1.76 to 2.51) | | 2.29 (1.82 to 2.88) | 2.19 (1.74 to 2.77) | | 1.93 (1.52 to 2.45) |  |
| Obesity, 30-34.9 kg/m^2^ | 4.02 (3.28 to 4.94) | | 4.33 (3.32 to 5.65) | 4.01 (3.07 to 5.24) | | 3.02 (2.25 to 4.04) |  |
| Severe obesity ≥35 kg/m^2^ | 6.03 (4.58 to 7.94) | | 4.99 (3.36 to 7.41) | 4.64 (3.12 to 6.90) | | 2.96 (1.91 to 4.58) |  |
|  |  | |  |  | |  |  |
| **Waist Circumference**, cases/controls | 212/116,543 | | 161/115,730 | 161/115,730 | | 161/115,730 |  |
| per cm | 1.05 (1.04 to 1.06) | | 1.05 (1.03 to 1.06) | 1.05 (1.03 to 1.06) | | 1.04 (1.02 to 1.05)^#^ |  |
| <88 cm(W) or <102 cm(M) | 1 (ref) | | 1 (ref) | 1 (ref) | | 1 (ref)^#^ |  |
| ≥88 cm(W) ≥102 cm(M) | 2.67 (2.02 to 3.52) | | 2.52 (1.83 to 3.45) | 2.52 (1.83 to 3.47) | | 1.92 (1.35 to 2.72) |  |
|  |  | |  |  | |  |  |
| **Systolic blood pressure**, cases/controls | 646/277,662 | | 490/275,899 | 490/275,899 | | 490/275,899 |  |
| per mmHg | 1.01 (1.006 to 1.02) | | 1.01 (1.002 to 1.01) | 1.01 (1.002 to 1.01) | | 1.00 (0.99 to 1.00) |  |
| <140 mmHg | 1 (ref) | | 1 (ref) | 1 (ref) | | 1 (ref) |  |
| ≥140 mmHg | 1.41 (1.18 to 1.68) | | 1.34 (1.09 to 1.65) | 1.32 (1.07 to 1.62) | | 0.99 (0.81 to 1.23) |  |
|  |  | |  |  | |  |  |
| **Diastolic blood pressure**, cases/controls | 646/277,662 | | 490/275,899 | 490/275,899 | | 490/275,899 |  |
| per mmHg | 1.02 (1.01 to 1.03) | | 1.02 (1.01 to 1.03) | 1.02 (1.01 to 1.03) | | 1.01 (0.996 to 1.01) |  |
| <90 mmHg | 1 (ref) | | 1 (ref) | 1 (ref) | | 1 (ref) |  |
| ≥90 mmHg | 1.48 (1.23 to 1.79) | | 1.47 (1.19 to 1.83) | 1.45 (1.17 to 1.81) | | 1.08 (0.86 to 1.35) |  |
| Supplement table 1 continues. |  | |  |  | |  |  |
| **Number of chronic diseases**, cases/controls | 857/278,598 | | 490/276,818 | 490/276,818 | | 490/276,818 |  |
| 0 | 1 (ref) | | 1 (ref) | 1 (ref) | | 1 (ref) |  |
| 1 | 1.94 (1.61 to 2.33) | | 1.90 (1.50 to 2.41) | 1.89 (1.49 to 2.39) | | 1.76 (1.39 to 2.22) |  |
| 2 | 3.47 (2.76 to 4.36) | | 2.58 (1.88 to 3.54) | 2.51 (1.83 to 3.45) | | 2.11 (1.54 to 2.90) |  |
| 3 | 5.80 (4.27 to 7.89) | | 3.83 (2.46 to 5.97) | 3.66 (2.35 to 5.71) | | 2.92 (1.87 to 4.56) |  |
| 4 to 5 | 10.7 (6.56 to 17.5) | | 8.15 (4.25 to 15.62) | 7.76 (4.04 to 14.90) | | 5.57 (2.88 to 10.77) |  |
|  |  | |  |  | |  |  |
| **Exercise habits**, cases/controls | 779/278,598 | | 490/276,818 | 490/276,818 | | 490/276,818 |  |
| Never/irregular | 1 (ref) | | 1 (ref) | 1 (ref) | | 1 (ref) |  |
| 1-2 times/week | 0.82 (0.69 to 0.97) | | 0.94 (0.76 to 1.16) | 1.00 (0.81 to 1.24) | | 1.12 (0.90 to 1.39) |  |
| ≥3 times/week | 0.75 (0.63 to 0.90) | | 0.88 (0.70 to 1.09) | 0.92 (0.74 to 1.15) | | 1.11 (0.88 to 1.40) |  |
|  |  | |  |  | |  |  |
| **Commute type**, cases/controls | 570/212,325 | | 425/211,147 | 426/211,147 | | 426/211,147 |  |
| Passive | 1 (ref) | | 1 (ref) | 1 (ref) | | 1 (ref) |  |
| Low dose (<20 min/day) | 0.94 (0.76 to 1.16) | | 0.98 (0.77 to 1.25) | 1.01 (0.79 to 1.28) | | 1.10 (0.86 to 1.41) |  |
| High dose (≥20 min/day) | 0.88 (0.65 to 1.20) | | 0.87 (0.61 to 1.25) | 0.90 (0.62 to 1.29) | | 1.02 (0.71 to 1.47) |  |
|  |  | |  |  | |  |  |
| **Physical work situation**, cases/controls | 592/251,733 | | 443/250,173 | 444/250,173 | | 444/250,173 |  |
| Mostly seated | 1 (ref) | | 1 (ref) | 1 (ref) | | 1 (ref) |  |
| Light activity | 1.18 (0.98 to 1.43) | | 1.19 (0.96 to 1.48) | 1.05 (0.84 to 1.31) | | 1.06 (0.85 to 1.33) |  |
| Moderate/heavy activity | 1.27 (1.01 to 1.59) | | 1.26 (0.97 to 1.63) | 1.05 (0.81 to 1.38) | | 1.06 (0.81 to 1.38) |  |
|  |  | |  |  | |  |  |
| **Diet habits**, cases/controls | 659/278,508 | | 490/276,766 | 491/276,766 | | 491/276,766 |  |
| Neutral/Good/Very good | 1 (ref) | | 1 (ref) | 1 (ref) | | 1 (ref) |  |
| Very poor/poor | 1.31 (0.95 to 1.80) | | 1.37 (0.96 to 1.96) | 1.30 (0.91 to 1.86) | | 1.01 (0.70 to 1.45) |  |
|  |  | |  |  | |  |  |
| **Alcohol habits**, cases/controls | 567/210,315 | | 423/209,170 | 423/209,170 | | 423/209,170 |  |
| Neutral/Good/Very good | 1 (ref) | | 1 (ref) | 1 (ref) | | 1 (ref) |  |
| Very poor/poor | 0.76 (0.49 to 1.17) | | 0.62 (0.36 to 1.09) | 0.67 (0.39 to 1.17) | | 0.66 (0.38 to 1.15) |  |
|  |  | |  |  | |  |  |
| **Daily smoker**, cases/controls | 659/278,598 | | 490/276,818 | 490/276,818 | | 490/276,818 |  |
| Never smoker/Occasionally | 1 (ref) | | 1 (ref) | 1 (ref) | | 1 (ref) |  |
| Daily smoker | 0.81 (0.61 to 1.07) | | 0.75 (0.53 to 1.06) | 0.63 (0.45 to 0.89) | | 0.61 (0.43 to 0.86) |  |
|  |  | |  |  | |  |  |
| **Stress Overall**, cases/controls | 659/278,517 | | 490/276,818 | 490/276,818 | | 490/276,818 |  |
| Sometimes/rarely/never | 1 (ref) | | 1 (ref) | 1 (ref) | | 1 (ref) |  |
| Very often/often | 1.24 (0.98 to 1.56) | | 1.32 (1.02 to 1.72) | 1.33 (1.02 to 1.72 | | 1.29 (0.99 to 1.67) |  |
| Model 1; adjusted for sex, age and performed year | |  | |  | |  | |
| Model 2; additionally adjusted for educational level, civil status and country of birth | | | | |  | |  |
| Model 3: additionally adjusted for estVO2max, BMI, number of chronic diseases, exercise habits, smoking, overall stress. | | | | | | |  |
| ^#^ adjusted as model 3, except for BMI | | |  |  | |  |  |

| **Supplement Table 2.** Odds ratio (95% CI) for sociodemographic predictors of severe COVID-19 in unmatched analyses | | | | |
| --- | --- | --- | --- | --- |
|  | **Non-complete data** | **Complete data for all adjusting variables** | | |
|  | Model 1-nc | Model 1-c | Model 2 | Model 2 |
|  | OR (95% CI) | OR (95% CI) | OR (95% CI) | OR (95% CI) |
| **Educational level**, cases/controls | 853/276947 | 490/276818 | 490/276818 | 490/276818 |
| University | 1 (ref) | 1 (ref) | 1 (ref) | 1 (ref) |
| High school/Voc. Education | 1.27 (1.07 to 1.52) | 1.31 (1.04 to 1.65) | 1.36 (1.08 to 1.72) | 1.18 (0.93 to 1.50) |
| Elementary school | 2.02 (1.61 to 2.54) | 2.03 (1.51 to 2.74) | 2.03 (1.50 to 2.73) | 1.65 (1.21 to 2.23) |
|  |  |  |  |  |
| **Civil status**, cases/controls | 856/278201 | 490/276818 | 490/276818 | 490/276818 |
| Married/co-habitat | 1 (ref) | 1 (ref) | 1 (ref) | 1 (ref) |
| Single/divorced/widower | 1.01 (0.87 to 1.16) | 0.97 (0.80 to 1.17) | 0.96 (0.80 to 1.16) | 0.96 (0.80 to 1.16) |
|  |  |  |  |  |
| **Country of birth**, cases/controls | 857/278598 | 490/276818 | 490/276818 | 490/276818 |
| Sweden | 1 (ref) | 1 (ref) | 1 (ref) | 1 (ref) |
| Else | 2.45 (2.07 to 2.90) | 2.51 (2.00 to 3.14) | 2.51 (2.00 to 3.15) | 2.45 (1.95 to 3.08) |
|  |  |  |  |  |
| **Occupation group**, cases/controls | 813/263319 | 471/262605 | 471/262605 | 471/262605 |
| White collar High skilled | 1 (ref) | 1 (ref) | 1 (ref) | 1 (ref) |
| White collar Low skilled | 1.41 (1.17 to 1.71) | 1.42 (1.13 to 1.82) | 1.18 (0.91 to 1.52) | 1.09 (0.84 to 1.42) |
| Blue collar High skilled | 1.34 (1.09 to 1.65) | 1.38 (1.04 to 1.82) | 1.11 (0.83 to 1.49) | 1.08 (0.81 to 1.45) |
| Blue collar Low skilled | 1.12 (0.91 to 1.37) | 1.29 (0.99 to 1.67) | 0.94 (0.70 to 1.24) | 0.87 (0.66 to 1.16) |
|  |  |  |  |  |
| **Income, thousands Swedish crowns**, cases/controls | 857/278598 | 490/276818 | 490/276818 | 490/276818 |
| Q4 | 1 (ref) | 1 (ref) | 1 (ref) | 1 (ref) |
| Q3-Q2 | 0.99 (0.84 to 1.17) | 1.10 (0.87 to 1.39) | 0.93 (0.73 to 1.17) | 0.88 (0.70 to 1.12) |
| Q1 | 1.19 (0.95 to 1.49) | 1.17 (0.87 to 1.58) | 0.88 (0.64 to 1.21) | 0.82 (0.59 to 1.12) |
| Model 1; adjusted for sex, age and performed year | |  |  |  |
| Model 2; additionally adjusted for educational level, civil status and country of birth | | | |  |
| Model 3: additionally adjusted for estVO2max, BMI, number of chronic diseases, exercise habits, smoking, overall stress. | | | | |

| **Supplement Table 3.** Indirect effects of socioeconomic factors on severe COVID-19 in un-matched analyses | | | | |  |  |
| --- | --- | --- | --- | --- | --- | --- |
|  | **BMI** | **CRF** | **Smoking^a^** | **Exercise** | **Stress** | **Total proportion mediated** |
|  | *ab* (95% HPD CI) | *ab* (95% HPD CI) | *ab* (95% HPD CI) | *ab* (95% HPD CI) | *ab* (95% HPD CI) |  |
| **Education** |  |  |  |  |  |  |
| High (0) vs low (1) | 0.043 (0.024 to 0.061) | 0.042 (0.032 to 0.051) | -0.168 (-0.266 to -0.074) | 0.001 (-0.005 to 0.007) | -0.004 (-0.010 to 0.002) |  |
| Proportion mediated | 0.106 | 0.103 | 0.413 | 0.002 | 0.010 | 0.634 |
| High (0) vs medium (1) | 0.029 (0.017 to 0.042) | 0.033 (0.032 to 0.051) | -0.100 (-0.157 to -0.043) | 0.000 (-0.002 to 0.003) | -0.002 (-0.006 to 0.001) |  |
| Proportion mediated | 0.148 | 0.168 | 0.510 | 0.000 | 0.010 | 0.837 |
| **Income** |  |  |  |  |  |  |
| Q4 (0) vs Q1 (1) | 0.033 (0.020 to 0.046) | 0.019 (0.032 to 0.051) | -0.090 (-0.147 to -0.034) | 0.001 (-0.003 to 0.004) | -0.002 (-0.006 to 0.002) |  |
| Proportion mediated | 0.172 | 0.099 | 0.469 | 0.005 | 0.010 | 0.755 |
| Q4 (0) vs Q2-Q3 (1) | 0.022 (0.013 to 0.031) | 0.017 (0.032 to 0.051) | -0.060 (-0.099 to -0.023) | 0.001 (-0.002 to 0.004) | -0.002 (-0.004 to 0.001) |  |
| Proportion mediated | 0.126 | 0.098 | 0.345 | 0.006 | 0.011 | 0.586 |
| **Occupation** |  |  |  |  |  |  |
| WCHS (0) vs BCLS (1) | 0.030 (0.018 to 0.043) | 0.034 (0.032 to 0.051) | -0.100 (-0.163 to -0.039) | 0.001 (-0.004 to 0.006) | -0.003 (-0.009 to 0.002) |  |
| Proportion mediated | 0.133 | 0.151 | 0.444 | 0.004 | 0.013 | 0.747 |
| WCHS (0) vs BCHS (1) | 0.026 (0.015 to 0.036) | 0.023 (0.032 to 0.051) | -0.090 (-0.148 to -0.036) | 0.001 (-0.005 to 0.008) | -0.003 (-0.008 to 0.002) |  |
| Proportion mediated | 0.137 | 0.121 | 0.474 | 0.005 | 0.016 | 0.753 |
| WCHS (0) vs WCLS (1) | 0.023 (0.014 to 0.033) | 0.025 (0.032 to 0.051) | -0.073 (-0.118 to -0.028) | 0.000 (-0.001 to 0.001) | -0.002 (-0.006 to 0.002) |  |
| Proportion mediated | 0.136 | 0.148 | 0.432 | 0.000 | 0.012 | 0.728 |
| Adjusted for sex, age, year HPA was performed, civil status, country of birth, and number of previous diseases as confounders of the exposure-mediator, exposure-outcome, and mediator-outcome relation. | | | | | |  |
| ^a^Smoking was coded as a binary variable (0 = never/seldom, 1 = daily smoker). | | |  |  |  |  |
| *ab*; indirect effect, HPD CI; highest posterior density credibility interval, BMI; body mass index, CRF; cardiorespiratory fitness, Q; quartile, WCHS; white-collar high-skilled, WCLS; white-collar low-skilled, BCLS; blue-collar low-skilled, BCHS; blue-collar high-skilled. | | | | | |  |
